# SIRT1-Related Signaling Pathways and Their Association With Bronchopulmonary Dysplasia

**DOI:** 10.3389/fmed.2021.595634

**Published:** 2021-02-22

**Authors:** Kun Yang, Wenbin Dong

**Affiliations:** Department of Newborn Medicine, The Affiliated Hospital of Southwest Medical University, Luzhou, China

**Keywords:** bronchopulmonary dysplasia, hyperoxia, oxidative stress, SIRT1, signaling pathways

## Abstract

Bronchopulmonary dysplasia (BPD) is a chronic and debilitating disease that can exert serious and overwhelming effects on the physical and mental health of premature infants, predominantly due to intractable short- and long-term complications. Oxidative stress is one of the most predominant causes of BPD. Hyperoxia activates a cascade of hazardous events, including mitochondrial dysfunction, uncontrolled inflammation, reduced autophagy, increased apoptosis, and the induction of fibrosis. These events may involve, to varying degrees, alterations in SIRT1 and its associated targets. In the present review, we describe SIRT1-related signaling pathways and their association with BPD. Our intention is to provide new insights into the molecular mechanisms that regulate BPD and identify potential therapeutic targets for this debilitating condition.

## Introduction

Revolutionary advances in perinatology have led to a considerable improvement in the survival rate of preterm infants. However, the incidence of bronchopulmonary dysplasia (BPD) is steadily increasing in infants of low gestational age and low birth weight. BPD imposes a heavy burden on families and society, partly due to its sophisticated pathogenesis and pathology, and partly because of the absence of a holistic definition for this disease, and the lack of effective treatment options (1). Oxidative stress is an established high-risk factor for BPD. An imbalance between oxidants and antioxidants creates an environment of oxidative stress in which reactive oxygen species (ROS) are produced in a partially reduced state (2). Premature infants are more prone to the induction of oxidative stress than full-term infants because of their immature lung function, inadequate pulmonary surfactant, and weak antioxidant enzyme system (3). To maintain healthy levels of oxygen saturation, it is important that these infants receive oxygen supplementation. However, this practice generates redundant forms of ROS, including superoxide anion radicals, hydrogen peroxide, and hydroxyl radicals (4); these act as virulence factors for proteins, carbohydrates, lipids, DNA, and RNA. In turn, these factors can activate apoptosis, leading to cellular dysfunction and structural disorders; they can also trigger tissue damage and epigenetic changes (2). These processes can culminate in the development of BPD, a condition that is characterized by alveolar epithelial and vascular endothelial cell inactivation, alveolar disorders, the accumulation of inflammatory factors, stromal cell proliferation, increased vascular permeability, and impaired capillary development (3).

**Figure 1:**
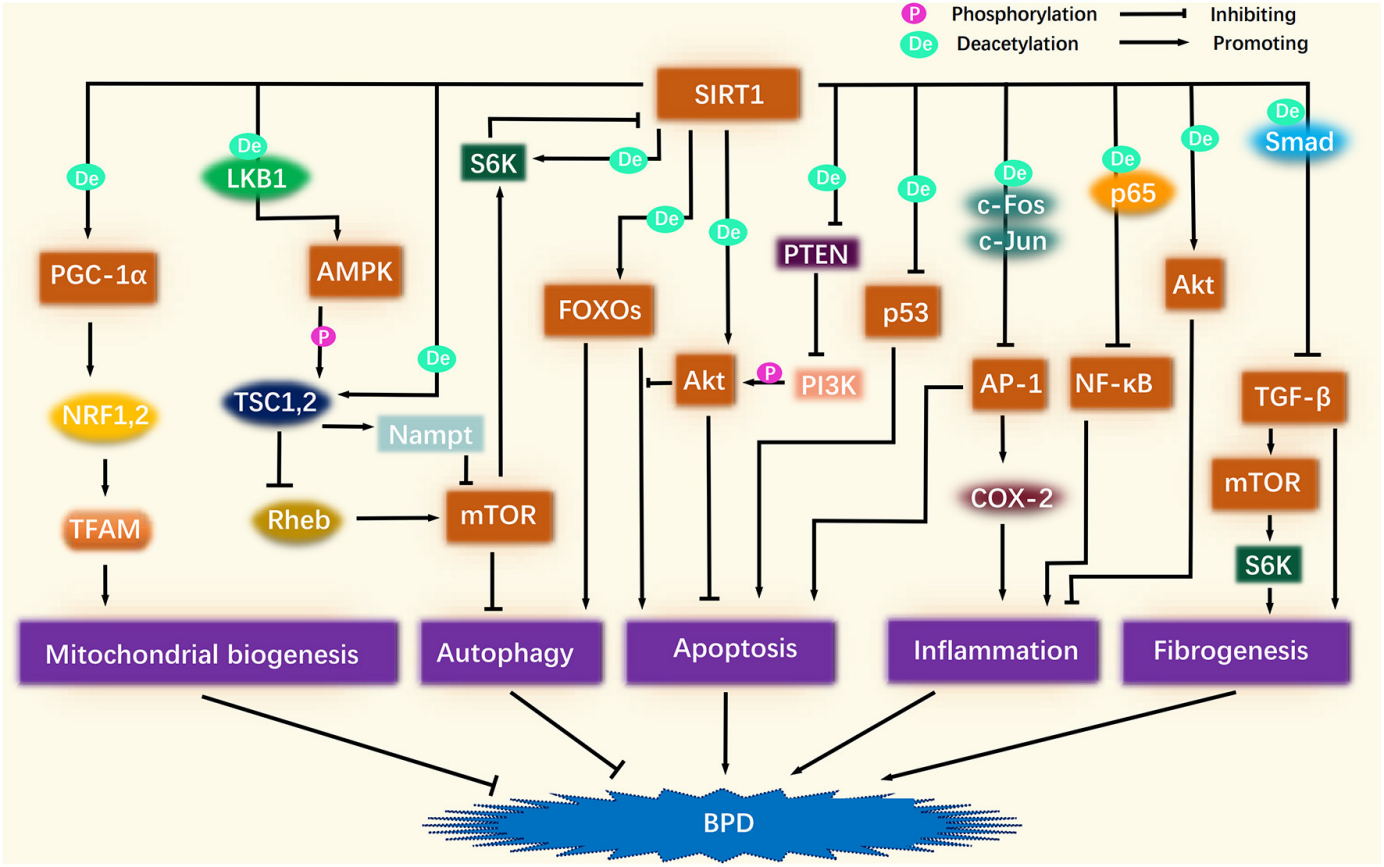
Association of SIRT1-related signaling pathways and BPD. SIRT1 deacetylation activates PGC-1α, which continues to activate NRF1/2 and TFAM in turn, thus promoting mitochondrial biogenesis to improve BPD. SIRT1 activates AMPK, and after AMPK phosphorylation activates TSC1/2, TSC1/2 activates Nampt, and inhibits Rheb, thereby inhibiting the role of mTOR in suppressing autophagy. A certain amount of autophagy plays a protective role against BPD. After SIRT1 activates FOXOs, FOXOs may promote autophagy along with apoptosis, but Akt inhibits the pro-apoptotic effect of FOXOs. SIRT1 directly activates Akt through deacetylation. SIRT1 also inhibits the inhibitory effect of PTEN on PI3K by deacetylating PTEN, thereby enhancing PI3K phosphorylation of Akt. The activation of Akt ameliorates BPD by inhibiting apoptosis and inflammation. SIRT1 also inhibits the pro-apoptotic effect of p53. SIRT1 inhibits the pro-inflammatory effects of NF-κB and AP-1 by deacetylating p65, c-Fos, and c-Jun. The pro-apoptotic effect of AP-1 is also inhibited by SIRT1. SIRT1 inhibits TGF-β by deacetylating Smad, thereby suppressing fibrogenesis. In addition, the inhibition of TGF-β attenuates its role in promoting mTOR, which is also linked to fibrogenesis.

Over recent years, an increasing body of evidence has come to support the association between BPD and alterations in silencing information regulator 2 related enzyme 1 (SIRT1) and its associated targets (5–7). As shown in Figure 1, SIRT1-related signaling pathways involve various biological processes, including mitochondrial biogenesis, autophagy, apoptosis, inflammation, and fibrogenesis, which potentially correlate intimately with BPD. However, this association has yet to be comprehensively reviewed. In the present review, we summarize the existing research relating to SIRT1 and other signaling pathways that may be related to BPD, including peroxisome proliferator-activated receptor γ co-activator 1α (PGC-1α), p53, protein kinase B (Akt), forkhead box O (FOXO), transforming growth factor-β (TGF-β), microRNA, nuclear factor kappa-B (NF-κB), activator protein-1 (AP-1), adenosine 5'-monophosphate-activated protein kinase (AMPK), and mammalian target of rapamycin (mTOR). We also propose potential therapeutic strategies for targeting SIRT1 to alleviate BPD.

## SIRT1 and BPD

SIRT1 belongs to the family of sirtuins proteins and is a nicotinamide adenine dinucleotide (NAD^+^)-dependent deacetylase that is distributed predominantly in cell nuclei. The *SIRT1* gene is located on chromosome 10q21.3 and encodes a 120 kD protein that contains two key domains, a highly conserved Rossmann fold, and a less conserved zinc finger structure and helix component (8). SIRT1 is engaged in a variety of cellular processes, including apoptosis, inflammation, mitochondrial function, and oxidative stress; SIRT1 carries out these functions by deacetylating histones H1, H3, and H4 and a range of non-histone proteins, including PGC-1α, NF-κB, p53, and FOXOs (9).

A vital consideration is that SIRT1 is closely associated with BPD. In a hyperoxia-induced mouse model of lung injury, lower levels of SIRT1 were shown to contribute to alveolar simplification and apoptosis (6). Other research has found that TNF-α, IL-1β, and NLRP3 inflammasome expression was upregulated in mouse lung microvascular endothelial cells upon knockdown of the *SIRT1* gene (10). In addition, Mody et al. (11) reported that the SIRT1 content of leukocytes that were acquired by aspiration from the trachea was markedly lower in BPD infants than in non-BPD infants. Furthermore, we found reduced levels of nuclear SIRT1, along with increased nucleocytoplasmic shuttling, in peripheral blood mononuclear cells (PBMCs) from BPD infants and preterm infants treated with different concentrations of oxygen, suggesting that hyperoxia or oxidative stress modified the activity and distribution of SIRT1 (12).

Indeed, oxidative stress probably mediates SIRT1 post-translational modifications, including phosphorylation, SUMOylation, S-nitrosylation, carbonylation, and S-glutathionylation, which reversibly or irreversibly affect SIRT1 activity (13). For example, the increased carbonylation of proteins has been shown to be involved in hyperoxia-induced lung injury in mice (14). SIRT1 levels and activity are reduced and SIRT1 protein degradation is accelerated in human lung epithelial cells under oxidative/carbonyl stress (15); these results contribute to the diminished ability of SIRT1 to acetylate p53, p65/RelA, and FOXO3, which causes inflammation, senescence, apoptosis, and endothelial dysfunction (13).

Furthermore, ROS possibly increases the expression of small ubiquitin-like modifier (SUMO)-specific protease 1 (SENP1), which attenuates SIRT1 deacetylation by de-SUMOylation of SIRT1 (7). Research also showed that the SUMOylation of SIRT1 increased its deacetylase activity and the stability of SIRT1 protein. Attenuated SUMOylation of SIRT1 increased stress-induced apoptosis, while enhanced SUMOylation of SIRT1 retarded cellular senescence (16, 17). In addition, SIRT1 interaction with SUMO1 and SUMO2/3 was significantly weakened in PBMCs of BPD patients, suggesting that the SUMOylation of SIRT1 is involved in the disease process (18).

Paradoxically, however, knockdown of the *SUMO1* gene increased pulmonary surfactant proteins, decreased glycogen content, and promoted lung differentiation in BPD models, illustrating silencing of the *SUMO1* gene protected against hyperoxia-induced lung injury in rats (19). Furthermore, SUMO1 and SUMO2/3 expression significantly increased in PBMCs of BPD infants, whereas SIRT1 expression markedly decreased (18), suggesting that more complex regulatory mechanisms regarding SIRT1 need to be further identified.

## SIRT1-Related Signaling Pathways and BPD

**Figure 2:**
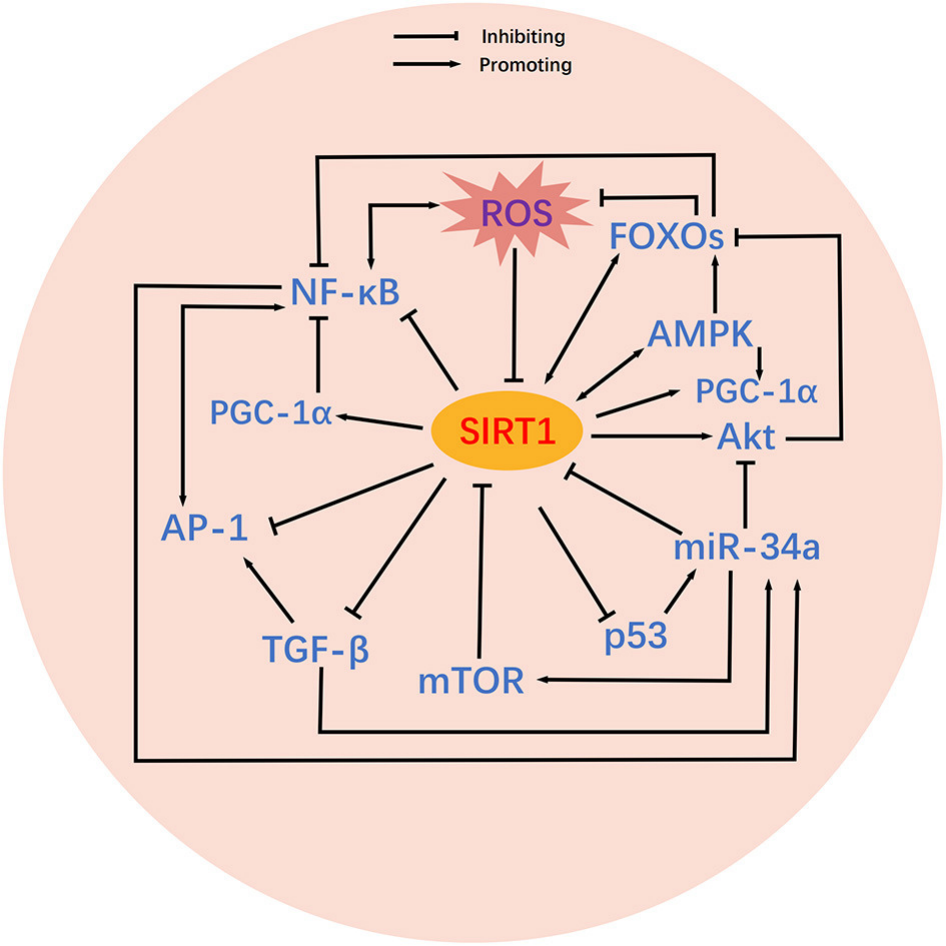
Crosstalk between SIRT1-related signaling pathways. Relatively complex crosstalk exists between SIRT1-related signaling pathways. SIRT1 activates or inhibits its downstream targets, including PGC-1α, p53, Akt, FOXOs, TGF-β, NF-κB, AP-1, AMPK, and mTOR. Mutual activating or inhibiting effects exist among the downstream targets of SIRT1. In addition, some downstream targets of SIRT1 also feedback regulate SIRT1 activity. MicroRNAs are also involved in the regulation of SIRT1 and its downstream targets. ROS inhibit the activity of SIRT1, thereby altering the associated signaling pathways.

It is possible that SIRT1 may act as an essential signaling hub for BPD. Risk factors for BPD such as hyperoxia can trigger changes in SIRT1 and thus initiate a chain of *domino effects*. As shown in Figure 2, these SIRT1-related signaling pathways are intricately intertwined, and can facilitate or inhibit each other to varying degrees and in different ways to influence the onset and development of BPD. Herein, we concentrate on the functions of these signaling pathways in BPD and their specific relationships with SIRT1.

### PGC-1α

Given that ROS are crucial enhancers of oxidative stress, and that mitochondria are the primary source of ROS (4), the role of mitochondrial function in BPD is receiving increasing amounts of research attention. In lung epithelial cells, hyperoxia exposure altered mitochondrial metabolism and dynamics, as evidenced by reduced basal and maximal respiration, diminished electron flow in respiratory chain complexes, and decreased utilization of metabolites such as glucose, glutamine, and fatty acids; there was also a significant change in mitochondrial morphology, distribution and mass. These results probably involved hyperoxia-induced alveolar simplification in mice (20). Conversely, enhanced fatty acid oxidation alleviated hyperoxia-induced endothelial cell apoptosis and lung injury in mice by attenuating ceramide synthesis and apoptosis (21).

In the BPD model, hyperoxia-exposed mice develop reduced alveolar counts with delayed development, thus mirroring the consequences induced by mitochondrial complex-I inhibitors (22). Similarly, mice under mechanical ventilation with prolonged high tidal volume showed the same impaired alveolarization and diminished expression of vascular endothelial growth factor as mice that had been insulted by an oxidative phosphorylation uncoupling agent (causing mitochondrial dysfunction) (23), thus suggesting that mitochondrial biogenesis exerts impact on lung development. Additionally, vascular endothelial of BPD-susceptible infants exhibited lower mitochondrial oxygen consumption, faster proton leakage, and significant mitochondrial ROS production and mitochondrial DNA (mtDNA) damage, thus suggesting that mitochondrial bioenergetics strongly correlates with BPD (24). MtDNA variants led to hyperoxic hypoalveolarization, pulmonary mechanical function, and mitochondrial redox dysfunction, further suggesting that mitochondria exert a profound impact on alveolar development (25).

Mechanistically, hyperoxia and mechanical ventilation can inhibit mitochondrial respiratory chain complexes, respiratory rate, membrane potential, and can also impede oxidative phosphorylation to reduce adenosine triphosphate (ATP) production, thereby causing atrophy or the abnormal growth of developing alveolar cells by depriving them of sufficient energy; eventually, this will lead to the BPD phenotype (26). Furthermore, ROS produced by mitochondria are also known to self-destruct, thus facilitating mitochondrial oxidative stress and accelerating lung injury (4).

Another important factor to consider is PGC-1α, a peroxisome proliferator-activated receptor gamma (PPARγ)-associated protein that is encoded by the *PPARGC1A* gene. PGC-1α is the master regulator of mitochondrial energy metabolism and quality control (27, 28). A previous study found that low expression levels of PGC-1α led to an increase in oxidative stress-induced mitochondrial dysfunction (29), and conversely, that the activation of PGC-1α mitigated lung injury from lipopolysaccharide (LPS) by enhancing mitochondrial biogenesis (30). The protective effect of PGC-1α on mitochondria is partly caused by the activation of antioxidant enzymes that attenuate the mitochondrial toxicity of ROS and prevent oxidative stress-induced apoptosis (8).

SIRT1 deacetylation activates PGC-1α, which subsequently activates nuclear respiratory factor 1 (NRF1) and NRF2. NRF1 and NRF2, as cell-important transcription factors, can regulate nuclear DNA (nDNA) to encode mitochondrial transcription factor A (TFAM). TFAM is transferred to mitochondria after nucleogenesis to control mtDNA transcription and replication (31), thus driving the production of mitochondrial respiratory enzymes and membrane proteins, thus promoting mitochondrial biogenesis (27, 32) (Figure 1). As an example, SIRT1 was shown to activate PGC-1α and NRF1 to alleviate hyperoxia-induced mitochondrial dysfunction in the lung epithelial cells, thereby reducing apoptosis (5). Moreover, activation of the SIRT1/PGC-1α/NRF/TFAM signaling pathway improved mitochondrial membrane potential, enhanced cytochrome c oxidase 1 (COX1) activity, and increased mtDNA and ATP content (33, 34). These factors may be involved in the protection against lung inflammation and oxidative stress in chronic obstructive pulmonary disease (COPD) mice (35).

Another key transcription factor is the nuclear factor (erythroid 2)-like (NF-E2), which is capable of modulating antioxidant response elements. There are 3 members of the NF-E2 family of proteins: NF-E2 related factor 1 (NF-E2-1), NF-E2-2, and NF-E2-3 (36). NF-E2-2, also a downstream molecule of SIRT1, is an essential protective factor against lung injury (37) and stimulates the expression of antioxidant genes and directly triggers the formation of antioxidant enzymes, such as heme oxygenase-1 (HO-1) and superoxide dismutase (SOD), to exert antioxidant effects (38). For instance, in a mouse model of lung growth retardation, hyperoxia caused the compensatory upregulation of NF-E2-2 and HO-1 in order to counteract oxidative stress (39). Furthermore, a deficiency of NF-E2-2 suppressed alveolar mitochondrial biogenesis and the transcription of anti-inflammatory factors (40), thus hampering alveolar maturation and exacerbating the hyperoxia-induced lung injury phenotype in mice (41).

On the one hand, SIRT1 activates NF-E2-2 by activating PGC-1α, thus reducing oxidative stress. For example, the activation of SIRT1/PGC-1α/NF-E2-2 pathway reduces chromium-induced lung injury in rats (42). On the other hand, SIRT1 can also deacetylate NF-E2-2 directly, thus enhancing its stability and ability to express SOD, catalase, glutathione, and HO-1 (43). It was found that activating SIRT1/NF-E2-2 delays aging by alleviating proteins oxidative damage and inflammatory factors such as IL-6 and TNF-α expression (44).

However, we should highlight the fact that although it has been established that the SIRT1/PGC-1α signaling pathway can promote mitochondrial biogenesis in other disease models, there is a clear lack of research to demonstrate a clear association between this pathway and BPD. Ongoing research in our own laboratory aims to address this shortfall.

### p53

The p53 protein is encoded by the *TP53* gene and has deservedly earned the nickname “*guardian of the genes*” on account of its contribution to genomic stability (45). Nevertheless, p53 is also associated with several detrimental biological processes in cells. For instance, p53 blocks cell cycle progression by activating its downstream target p21 (an inhibitor of cyclin-dependent kinases) (46, 47). P53 can also mediate several proteins (e.g., Bax/Bcl2, Fas/Apol, insulin-like growth factor binding protein 3, and tumor necrosis factor receptors) (8) to induce apoptosis; these processes may play a critical role in triggering BPD.

Previous research, carried out in baboons, showed that oxygen supplementation can induce high expression levels of p53 and p21 and thus encourage apoptosis in lung cells, thus leading to the inhibition of cell growth and the promotion of a BPD phenotype (47). In addition, hyperoxia disrupts lung development and repair by activating p53 and p21 to inhibit the activity of vascular endothelial growth factor (46), and to facilitate the senescence of both airway smooth muscle cells (48) and lung fibroblasts (49). The other direction to consider is that the p53/p21 signaling axis also modulates senescence. Research has shown that the expression of the senescence markers p21 and p16 increased in type II alveolar epithelial cells (AECII) from fibrotic lung disease, while silencing p53 attenuated cellular senescence by inhibiting p21 (50). Enhanced β-galactosidase activity in lung tissue from COPD patients represents the presence of senescence, while low levels of SIRT1 and FOXO3a, and high levels of p53 and p21 are probably involved in senescence process (51). Furthermore, post-prandial triglyceride-rich lipoproteins attenuate SIRT1 deacetylating p53 by increasing oxidative stress, which leads to the upregulation of acetylated p53 and p21 expression and finally to premature senescence of adipose-derived mesenchymal stem cells (52). These data suggest that p53 and p21 may be essential molecular factors in the initiation and development of BPD. The detection of p53 in ROS-damaged DNA may represent the beginning of hyperoxia-induced lung dysplasia (46, 47).

SIRT1 inhibits the transcriptional activity of p53 by deacetylating the p53 C-terminal lysine-382 residue and altering the conformation of p53 to reduce its ability to bind to DNA (Figure 1). Furthermore, SIRT1 diminishes the expression of the p21 and p53-regulated apoptotic factors to promote cell repair and survival (53). The reduced levels of histone deacetylase activity contributes to the upregulation of p53 and p21, which are involved in cell cycle blockade and epigenetic alterations in alveolar cells during hyperoxia in mice (54). In contrast, the SIRT1 agonist SRT1720 was shown to protect lung function and reduce lung injury in a mouse model of COPD by down-regulating the levels of p53 to reduce apoptosis in AECII (55). Furthermore, resveratrol has been shown to activate SIRT1 to inhibit DNA damage by p53, thus protecting against architecture disorders in the lungs caused by lung aging (56) and attenuating hyperoxia-induced apoptosis and alveolar simplification in neonatal rats (6).

### Akt and FOXOs

Akt, a protein that belongs to the AGC family, contains 3 components (a pleckstrin homology domain, a central kinase, and a regulatory domain) and has 3 isoforms: Akt1, Akt2, and Akt3. An essential upstream activator of Akt is phosphatidylinositol 3 kinase (PI3K); phosphatase and tensin homolog deleted on chromosome 10 (PTEN) has been shown to reduce Akt activity by dephosphorylating PI3K (57). Akt, as a survival protein, plays an incredibly important role in protecting cell survival and proliferation under hyperoxia (58).

Previous research showed that a rat model of hyperoxia-induced lung injury exhibited reduced levels of Akt activity, and that activation of Akt dramatically reduced apoptosis and increased the survival of lung epithelial cells (59). In addition, the transfection of Akt was shown to improve alveolar development and attenuate pulmonary arterial hypertension in BPD rats (59). Conversely, the suppression of PI3K/Akt, or knockdown of the *Akt1* gene, was shown to exacerbate lung injury and lung inflammation in mice by reducing the levels of Nrf2 (60). It is not difficult to infer from these data that Akt is an essential protective factor for BPD. Paradoxically, however, Reddy et al. (60) reported that PI3K/Akt also promoted lung injury in the late phases of hyperoxia and did so in a non-Nrf2-dependent manner. One possible explanation is that this is a manifestation of decompensation, or that other factors such as inflammation and oxidants may alter the direction of the PI3K/Akt signaling pathway. Consequently, it is evident that the regulation of such signaling pathways in a host is extremely precise and may exert dramatically divergent outcomes over both time and space.

Once activated by ROS, PI3K can phosphorylate Akt which then goes on to phosphorylate downstream targets such as BAD, FOXO3a, IκB kinase (IKK)-kinase, and murine double minute 2 (MDM2), thus participating in the regulation of apoptosis, inflammation, and oxidative stress (8, 58). In addition, the activation of PI3K/Akt may alleviate hyperoxia-induced oxidative damage in human pulmonary alveolar epithelial cells by up-regulating HO-1 (61).

SIRT1 is known to activate Akt by deacetylation. Specifically, SIRT1 reinforces the binding of phosphatidylinositol 3,4,5-trisphosphate (PIP3) to the pleckstrin homology domain and the phosphorylation of Akt at Thr308 and Ser473; these are critical steps in the activation of Akt (62) (Figure 1). Previous research showed that the inhibition of SIRT1 led to the suppression of proliferation and survival in neuronal SH-SY5Y cells by attenuating the PI3K/Akt pathway (63). In contrast, the activation of SIRT1 was shown to reduce the inhibitory effect of PTEN on Akt, thus reducing methamphetamine-induced oxidative stress and apoptosis in alveolar epithelial cells and protecting alveolar epithelial permeability and barrier function (64). In addition, resveratrol has been shown to rejuvenate AECII by activating Akt and MDM2, and by inhibiting p53 and PTEN; collectively, these processes improved the lung function and structure in senescent mice (56).

FOXO belongs to a subfamily of FOX transcription factors. Mammals express FOXO1, FOXO3, FOXO4, and FOXO6; FOXO1 and FOXO3 are closely related to cell proliferation, oxidative stress, autophagy, apoptosis, and metabolism (65). As transcription factors, FOXO proteins may exert both positive and negative effects in BPD. On the one hand, FOXOs promote autophagy by activating autophagy genes, by binding to autophagy proteins, and by altering epigenetic status (66). FOXOs can also counteract ROS by enhancing the expression of antioxidant proteins, such as SOD, catalase, thioredoxin, and glutathione (67). On the other hand, FOXOs can also promote apoptosis by blocking the cell cycle (68). In addition, previous research has shown that ROS can mediate the transcription and regulation of FOXOs, as well as post-translational modifications, subcellular localization, and protein synthesis (67).

It is important to note that Akt phosphorylates FOXOs to inhibit their pro-apoptotic activity, and that SIRT1 deacetylates FOXOs to enhance their autophagic and antioxidant effects. Akt reduces the binding of FOXO1 and FOXO3 to apoptotic genes by reducing their transcriptional activity and by inducing nucleoplasmic translocation (69). Advantageously, the ability of Akt to phosphorylate FOXO1 and FOXO3 is enhanced after activation by SIRT1 deacetylation. A previous study confirmed that a mouse model of hyperoxia-induced acute lung injury showed diminished expression of phosphorylated Akt and FOXO1 (70). This may contribute to increased levels of apoptosis and thus participate in the BPD phenotype. This conjecture is supported by the fact that suppression of PI3K/Akt reduces the expression of phosphorylated FOXO3a, thus increasing the levels of Bim and Bax and reducing the levels of Bcl-2 and CyclinD1 to promote apoptosis in AECII (71).

In contrast, activation of the Akt phosphorylation FOXO1 signaling pathway was shown to enable the regeneration of lung endothelial and epithelial cells in a rat model of LPS-induced lung injury (68). In a model of BPD, the activation of Akt/FOXO3 was also involved in the development of alveoli and pulmonary vasculature via the inhibition of apoptosis (69).

SIRT1 is known to enhance the autophagic and antioxidant capacity of FOXO1 and FOXO3 via deacetylation. FOXO1 and FOXO3 may also facilitate the expression of SIRT1, thus creating a form of self-feedback (72, 73) (Figure 1). For instance, resveratrol has been shown to activate FOXOs to resist oxidative stress in C2C12 cells by increasing levels of SOD and by decreasing ROS levels (74). Furthermore, when exposed to cigarettes, SIRT1 agonists were unable to reverse the high levels of lipid peroxidation products and the low expression of antioxidants in the lung tissue of *FOXO3* knockout mice, but did so in wild-type mice, thus suggesting that FOXO3 can also mediate the effects of SIRT1 (75). Surprisingly, the deacetylation of FOXO3 by SIRT1 was also shown to be involved in the inhibition of apoptosis in the AECII of the model COPD (55), thus suggesting that FOXOs may have distinct effects that are dictated by different modifications.

However, it is important to note that only very limited research has been undertaken on the role of FOXOs in BPD. In addition, it is possible that the role of FOXOs may depend heavily on the function of its upstream targets. Therefore, further research is urgently needed to investigate the mechanisms that link FOXOs with BPD.

### TGF-β

TGF-β belongs to the superfamily of secreted growth factors, which also includes a range of other members, including bone morphogenetic proteins, activins, and growth factors (76). TGF-β ligands (such as TGF-β1, TGF-β2, and TGF-β3) bind to TβR II and TβR I receptors in the cell membrane to form complexes, thus initiating the TGF-β signaling pathway. TβR I is activated by the phosphorylation of TβR II and then activates Smad2 and Smad3, which are then bound to Smad4 for translocation to the nucleus and participate in gene transcription processes such as cell proliferation, development, and apoptosis (76, 77).

TGF-β is an influential regulator of lung development (78), but also represents a *double-edged sword*. The overexpression of TGF-β, and prolonged changes in the levels of TGF-β, will cause a series of detrimental effects on the lungs, including reduced gas exchange and lung function, poor alveolar development and angiogenesis, the emergence of pulmonary fibrosis, and BPD (76, 79). Previous research showed that the expression of TGF-β1 rose significantly with time in the lung tissue of mice exposed to hyperoxia (80). When transfected with the *TGF-*β*1* gene, experimental mice replicated the BPD phenotype (81). In monkeys, increased levels of TGF-β1 caused uncontrolled levels of cell proliferation and collagen deposition, thus leading to pulmonary fibrosis and lung dysplasia (82). Moreover, over-activation of the TGF-β signaling pathway has been shown to be involved in the abnormal development of alveoli and branches in hyperoxia by inducing DNA methylation and other modifications (83), thus suggesting that TGF-β can affect lung development in different ways.

Conversely, the antagonization of TGF-β signals prevented overexpression of the extracellular matrix and its remodeling proteins, reduced elastin production, and corrected its abnormal distribution and heterogeneous accumulation, thus alleviating hyperoxia-induced alveolar simplification, morphological defects in alveolar cells, and apoptosis in mice (78, 79). In addition, inhibition of TGF-β also promoted alveolar formation, microvascular development, and body weight gain, in the damaged lungs of mice under hyperoxia (84).

The inhibitory effect of SIRT1 on TGF-β may occur via the deacetylation of Smad2, Smad3, and Smad4 (85–87); these are critical downstream targets of TGF-β and are intimately associated with the formation of tissue fibrosis (88) (Figure 1). A previous study found that resveratrol attenuated LPS-induced epithelial-mesenchymal transformation and lung fibrosis by weakening TGF-β1/Smad signaling (89). Furthermore, resveratrol has been shown to effectively downregulate TGF-β expression in bronchoalveolar lavage fluid (BALF) in mice models of COPD and bleomycin-induced lung fibrosis (90, 91).

### miR-34a

MicroRNA (miRNA) is a class of short (21–24 nucleotides in length), relatively conserved, single-stranded RNAs that are encoded by endogenous genes. Following transcription miRNAs are involved in a variety of biological processes, including cell differentiation, development, proliferation, angiogenesis, and inflammation; these effects occur in response to miRNAs exerting regulatory effects on the expression levels of mRNA and protein (92, 93). In a previous study, Bhaskaran et al. (93) reported increased expression levels of miR-21 and miR-34a in the lung tissue of mice exposed to hyperoxia; in contrast, levels of miR-342, miR-335, miR-150, miR-126^*^, and miR-151^*^, were all reduced. A meta-analysis also showed that miRNA-21, miRNA-34a, miRNA-431, and Let-7f, were up-regulated in BPD, while miRNA-335 was down-regulated (92). Predictably, these BPD-associated miRNAs may represent a new perspective to the pathogenesis of this disease.

It is important to highlight that of all miRNAs, miR-34a is known to show the strongest association with BPD. For example, Syed et al. (94) reported an alleviation of the BPD phenotype in mice with an overall or AECII specific deletion of miR-34a and that this was associated with enhanced tolerance to hyperoxia and reduced levels of inflammatory infiltration and lung injury. The overexpression of miR-34a, however, evoked impairment of alveolarization and angiogenesis in mice held in room air, presumably because hyperoxia promoted the miR-34a-induced inhibition of the protective effects of angiopoietin-1 (Ang1) and its receptor, Tie2, on the lung, thereby inducing apoptosis (94). Importantly, these authors detected increased levels of miR-34a in tracheal aspirates and samples of lung tissue from children with BPD (94), thus indicating that miR-34a is a valuable predictor for BPD.

Furthermore, when exposed to hyperoxia and miR-34a, platelet-derived growth factor receptor α-expressing myofibroblasts, which are associated with alveolar formation, produced an increase in defective elastin, thus resulting in the simplification of alveoli and thickened intervals. In contrast, a deficiency of miR-34a in BPD mice, led to the promotion of alveolar development (95).

Mechanistically, hyperoxia stimulates p53 to activate miR-34a, which then interferes with a series of signals to induce the BPD phenotype (96). In particular, miR-34a inhibits SIRT1 to augment the pro-apoptosis of p53, which in turn provides positive feedback to miR-34a to further reduce the expression of SIRT1 (96) (Figure 2), thus facilitating bleomycin-induced lung epithelial injury and fibrosis in mice (97). Furthermore, miR-34a is known to promote the negative regulation of autophagy by mTOR (96) and inhibits Ang1/Tie2 phosphorylation of Akt to induce cell death (94). MiR-34a can also be up-regulated by TGF-β, thus enhancing the inhibition of SIRT1 (94).

In contrast, the inactivation of miR-34a can enhance the ability of SIRT1 to counteract ROS and apoptosis (98). The activation of SIRT1 alleviated miR-34a-mediated endothelial dysfunction, inflammatory infiltration, and vascular damage, in a mouse model of LPS-induced lung injury (99).

### NF-κB and AP-1

NF-κB, initially discovered in the nuclei of B lymphocytes, is a transcription factor that binds to a specific sequence of the immunoglobulin κ light chain (100). NF-κB contains 5 subunits (p65/RelA, Rel B, cRel, p50, and p52), which form homologous or heterologous dimers with each other and are among the most significant regulators of inflammation and redox responses (100). In the early stages of alveolar development, NF-κB exerts a protective effect on the lung by reducing inflammation via the inhibition of macrophage inflammatory protein-2 (MIP-2) expression (101). Nevertheless, excessive inflammatory irritation and oxidative stress can shift the action of NF-κB toward a pathological state; this may represent one of the most critical steps in the initiation of BPD. Once aberrantly activated by IL-1β, TNF-α, or ROS, NF-κB translocates to the nucleus (102) and cooperates with other transcription factors, such as AP-1, signal transducer and activator of transcription 3 (STAT3), early growth response protein 1 (EGR-1), and specificity protein 1 (SP-1), to produce large amounts of chemokines, adhesion molecules, and pro-inflammatory factors, such as TNF-α, IL-1, IL-6, MIP-1 (100), thus triggering an uncontrolled inflammatory response. These processes may trigger the abnormal development of alveoli and the pulmonary blood vessels.

Previous research found that the levels of NF-κB in tracheal lavage fluid were excessively high in BPD patients (103). Other research detected the abnormal expression of NF-κB in mechanically ventilated alveolar macrophages in preterm infants (104). The activation of NF-κB promotes the secretion of IL-1β by lung macrophages; these interfere with the development of airways in the canalicular and saccular phases, thus causing the BPD alveolar phenotype (105). NF-κB has also been shown to significantly inhibit the effect of fibroblast growth factor-10, a critical factor in molding the typical morphology of the lung, on airway lengthening and branching (106). In addition, NF-κB has been shown to be involved in the apoptosis of lung mesenchymal cells under hyperoxia conditions (107). The excessive nuclear aggregation of NF-κBp65 is now a known predictor of BPD severity as this regulates the proliferative capacity of mesenchymal stromal cells (108).

NF-κB activity is regulated by post-transcriptional phosphorylation, acetylation, and by methylation modifications. SIRT1 is also known to mitigate the pro-inflammatory effects of NF-κB by deacetylating p65 (109) (Figure 1). Because of this, *SIRT1* knockout sepsis mice appear to have active NF-κB, STAT3, and extracellular signal-regulated kinase (ERK) 1/2, thus providing the lungs with an unusually strong inflammatory signal by upregulating a range of pro-inflammatory mediators, including IL-ip, IL-6, and TNF-α (110). Furthermore, bleomycin reduced the expression levels of SIRT1 in the BALF of mice with pulmonary fibrosis; the reduced levels of SIRT1 subsequently elevated the levels of NF-κBp65 and promoted lung inflammation (111).

Conversely, the activation of SIRT1 attenuated LPS-induced myeloperoxidase activity and the expression of TNF-α, IL-1β, and IL-6, in the lungs of mice by inhibiting NF-κB activity (112). In a similar manner, SIRT1 alleviated sepsis-associated lung inflammation in mice by inhibiting NF-κB, STAT3, ERK1, and p38 (113). Furthermore, SIRT1 was shown to inhibit NF-κB acetylation to reduce the oxidative stress and apoptosis caused by lung injury in mice (114). However, the interaction of SIRT1 with NF-κB is not unidirectional. NF-κB is known to inhibit SIRT1 by enhancing the expression of miR-34a and by promoting the production of ROS and nitric oxide radicals (115).

Notably, AP-1 exerts a synergistic effect with NF-κB to co-activate the inflammatory response through direct contact, or by sharing the promoter sequence of pro-inflammatory genes (100). AP-1 is a member of the basic region/leucine zipper protein family; this is the common name for several transcription factors, including Jun, Fos, ATF, MAF, and their subfamilies (116). AP-1 is involved in a number of biological processes, including cell growth, differentiation, and apoptosis, and can be activated by multiple signals, including chemokines, growth factors, cytokines, hormones, and pathogens, through several signaling pathways represented by mitogen-activated protein kinase (MAPK). Stimulatory factors activate MAPK kinases, which can then phosphorylate MAPK family members such as ERK1/2, c-Jun N-terminal kinase (JNK), and p38; these can then activate AP-1 (100, 116). Furthermore, AP-1 is also activated by TGF-β by the significant elevation of the expression levels of c-Fos and c-Jun (117).

AP-1 is also an essential regulator of the oxidative stress response; this is due to its sensitivity to the regulation of antioxidant genes and stimulation by ROS (100, 118). Previous research revealed that activation of AP-1 and its upstream targets, JNK and p38MAPK, are involved in hyperoxia-induced epithelial cell death in lungs of mice, and mitochondrial damage (119); these effects may be caused by the upregulation of AP-1 by redundant ROS- activated JNK and the subsequent activation of IL-8 which can cause damage to lung epithelial cells (120). In addition, the activation of AP-1 and NF-κB by hyperoxia promotes the expression of TNF-α and IL-1β; these processes are also involved in the process of lung injury (121).

SIRT1 deacetylates c-Fos and c-Jun to inhibit the ability of AP-1 to transcribe cyclooxygenase-2 and monocyte chemoattractant protein-1 (MCP-1) (122, 123) (Figure 1). High levels of cyclooxygenase-2 and MCP-1 are also known to promote lung inflammation in a mouse model of hyperoxia-induced lung injury (124), while the inhibition of cyclooxygenase-2 partially attenuates the BPD phenotype (125). In addition, Wang et al. (117) reported that resveratrol attenuated bleomycin-induced lung fibrosis in mice by inhibiting the MAPK/AP-1 pathway, possibly because resveratrol reduced the levels of c-Fos and c-Jun, thereby inhibiting the miR-21-induced activation of TGF-β/Smad signaling.

### AMPK and mTOR

Stable autophagy is a protective measure employed by cells that involves the transportation of harmful substances, such as oxidized proteins and lipids, through autophagic vesicles to lysosomes for self-degradation (8).

Autophagy is essential for the natural shaping of lungs, and levels of postnatal basal autophagic activity may, in turn, be regulated by alveolar development (126, 127). However, the over-activation or inhibition of autophagy, and the retention of autophagosomes, is likely to trigger an imbalance in autophagy, thus inducing harmful events such as inflammation and apoptosis. Neonatal mice exposed to hyperoxia showed impaired autophagy, resulting in thickened alveolar septa, increased apoptosis, and disrupted alveolar development; however, the addition of an autophagy inducer ameliorated these manifestations (128). Furthermore, autophagy-deficient mice showed increased susceptibility to hyperoxia-induced lung injury, with the emergence of high expression levels of inflammatory mediators and a severe BPD phenotype (127), thus indicating that a certain amount of autophagy plays a crucial protective role in BPD.

AMPK and mTORC1 are the two primary modulatory molecules of autophagy in hyperoxia-induced lung injury and can positively and negatively regulate autophagy, respectively (127). MTOR is a serine/threonine-protein kinase and contains two multiprotein complexes: mTORC1 and mTORC2. Of these, mTORC1, is the most closely related to autophagy, and includes several components: mTOR, regulatory associated protein of mTOR (RAPTOR), MLST8, PRAS40, and DEPTOR (129). The inhibition of RAPTOR was found to alleviate the hyperoxia-induced apoptosis of lung epithelial cells and the disruption of lung tissue structure in mice by enhancing autophagy (130). MTORC1 is known to negatively regulate autophagy by inhibiting unc-51 like kinase 1 (ULK1) and phosphoinositide 3-kinase class III complex, thus blocking lysosomal biogenesis-related genes, activating acetyltransferase p300, and producing substrate death-associated protein 1 (129).

In addition, mTOR appears to be associated with cell proliferation and lung fibrosis. It has been shown that the suppression of mTOR allows neonatal tracheal aspirate-derived basal-like cells, associated with lung development, to expand long-term and promote their differentiation into functional airway epithelial cells (131). Furthermore, TGF-β is known to increase the activity of mTOR and ribosomal S6 kinase (p70S6K), a fibrosis-related factor (111), thereby promoting lung fibrosis. The inhibition of mTOR, however, was shown to attenuate the fibrosis of lung tissue and lung fibroblasts in mice under hyperoxia by downregulating the levels of its downstream targets (p70S6K and 4EBP1) and by decreasing the expression of TGF-β to diminish collagen deposition (132). Surprisingly, mTOR and p70S6, which are dependent on Akt activation, are involved in cell survival by increasing cellular uptake and the utilization of glucose under early exposure to hyperoxia, thus suggesting that mTOR also exerts a protective effect on the lung (58). These findings indicate that these molecular actions are not invariant but are highly influenced by activators and may exhibit opposite effects in different stages of the disease.

In other research, Yeganeh et al. (126) reported that during the development of the lungs in mice, AMPK levels appear to represent autophagic activity, as suppression of AMPK replicates the autophagy inhibitor-induced damage to lung branches. Both mouse and baboon BPD models exhibit AMPK underactivity and hyperactivity of mTORC1; the consequence of these actions is the impairment of autophagy, thus leading to lung injury (127). In contrast, activation of the AMPK/mTOR pathway enhanced autophagic flux and reduced the generation of intracellular ROS, thus alleviating endothelial dysfunction (133). Collectively, these data corroborate the positive effect of AMPK on autophagy.

From a mechanistic point-of-view, AMPK (as an AMP-dependent serine/threonine-protein kinase) promotes autophagy by phosphorylating tuberous sclerosis protein (TSC) to inactivate ras homolog enriched in brain (Rheb), and by phosphorylating RAPTOR to weaken its interaction with the mTORC1 substrate, respectively. In addition, AMPK also activates the ULK1 complex and FOXO3, acting on late endosomes/lysosomes and other pathways to positively regulate autophagy (134).

Interestingly, AMPK also exhibits essential roles in cellular energy metabolism, inflammation, and apoptosis. AMPK strengthens the deacetylation of PGC-1α by SIRT1 (9) and can directly phosphorylate PGC-1α (27) to upgrade mitochondrial biogenesis. Furthermore, AMPK inhibits the pro-inflammatory effects of NF-κB by activating SIRT1 to boost the deacetylation of RelA/p65, activating FOXO3a to inhibit the nuclear translocation of RelA/p65 (115), and activating PGC-1α to reduce p65 phosphorylation and transcriptional activity (100).

SIRT1 is also a vital regulator of autophagy and is known to influence autophagy via crosstalk with AMPK and mTOR (Figure 1). SIRT1 can also promote the maturation of autophagosomes by deacetylating the autophagy regulator Beclin1 and microtubule-associated protein 1 light chain 3 (135). The intrinsic relationship between SIRT1 and AMPK is bidirectional and positively oriented. On the one hand, SIRT1 up-regulates AMPK by deacetylating liver kinase B1 (LKB1) and by activating Ca2+/calmodulin-dependent protein kinase kinase β channels (8). For example, SIRT1/AMPK signaling exhibits anti-aging effects in human lung epithelial cells by promoting autophagy and reducing ROS and mitochondrial superoxide (136). On the other hand, AMPK activates SIRT1 by elevating NAD^+^ content and by increasing the NAD/NADH ratio (137). For instance, activation of the AMPK/SIRT1 signaling pathway was shown to attenuate lung inflammation and apoptosis in a rat model of sepsis (138) and ameliorated LPS-induced impairment of alveolar epithelial barrier function (139).

The crosstalk between SIRT1 and mTOR is bidirectional and negatively oriented. SIRT1 deacetylates TSC1/TSC2; this leads to the inactivation of Rheb and the inhibition of mTOR (140). The interaction between SIRT1 and TSC2 also promotes high expression levels of nicotinamide phosphoribosyltransferase (NAMPT), which induces autophagy via the inhibition of mTORC1 (135). In turn, mTOR directly phosphorylates SIRT1 to reduce its deacetylase activity. In addition, SIRT1 activates the mTORC1 downstream target S6K1 by deacetylation; S6K1 can also inhibit SIRT1 by mimicking the activity of mTORC1 (135).

## Conclusion and Future Prospects

BPD is a prevalent complication of prematurity. The pathogenesis and pathology of BPD is complex and can be confusing. However, research is now providing significant insight into this condition. SIRT1 is considered to be a long-lived protein and has been receiving increasing levels of attention over recent years with regards to BPD. Alterations in SIRT1, and its associated targets, may be involved in the molecular events associated with the onset and development of BPD. The profound association between the SIRT1-related signaling network in BPD may contribute to the development of new approaches for the treatment of BPD.

In this review, we described the role of SIRT1 and BPD-associated signaling pathways, including PGC-1α, p53, Akt, FOXOs, TGF-β, microRNA, NF-κB, AP-1, AMPK, and mTOR. These factors are activated simultaneously or sequentially in response factors that are considered to be high risk for BPD, including hyperoxia and mechanical ventilation, thereby inhibiting or promoting the formation of BPD to varying degrees. The upregulation of SIRT1 may drive these signaling pathways in a favorable direction. Hence, small-molecule agonists of SIRT1, such as resveratrol, are expected to become an effective therapy for BPD.

A range of small molecules are known to activate SIRT1; the first of these to be discovered was resveratrol, a natural agonist of SIRT1 (9). Resveratrol is a defensin that is present in the roots, stems, leaves, and pericarp, of a diverse range of plants that can sense danger signals (141, 142). Resveratrol is known for its wide range of targets (e.g., transcription factors, cytokines, and several enzymes) and its diverse range of physiological effects (e.g., anti-platelet aggregation, antioxidant, and anti-inflammatory) (142). Resveratrol has been demonstrated to enhance mitochondrial biogenesis (143), ameliorate the endothelial barrier (144), promote autophagy (145), boost antioxidant capacity (12), attenuate apoptosis (6), and reduce inflammation (146) by activating SIRT1. In addition, synthetic SRT1720, SRT2183, and SRT1460, are also commonly used agonists of SIRT1 (9). Based on the critical function of SIRT1 in a wide range of diseases, it is evident that targeted SIRT1 therapy may lead to favorable clinical outcomes. It is imperative that we develop a new generation of SIRT1 agonists.

However, we should highlight that the regulatory processes associated with SIRT1 signaling networks are intricate and elaborate. Furthermore, their specific function may vary instantaneously depending on their upstream and downstream targets, time and space. Therefore, further experimental and clinical data are now needed to support a more precise role for these signals in BPD, as well as long-term follow-up results to validate the value of targeted SIRT1 therapy for BPD.

In summary, gaining an increased understanding of the SIRT1 signaling network will significantly facilitate our ability to intervene with the pathogenic processes underlying BPD and will create novel perspectives for targeting SIRT1 for BPD treatment.

## Author Contributions

KY wrote the manuscript. WD audited the manuscript. Both authors contributed to the article and approved the submitted version.

## Conflict of Interest

The authors declare that the research was conducted in the absence of any commercial or financial relationships that could be construed as a potential conflict of interest.

## Abbreviations

SIRT1: silencing information regulator 2 related enzyme 1
PGC-1α: peroxisome proliferator-activated receptor γ co-activator 1α
Akt: protein kinase B
FOXO: forkhead box O
TGF-β: transforming growth factor-β
NF-κB: nuclear factor kappa-B
AP-1: activator protein-1
AMPK: adenosine 5′-monophosphate-activated protein kinase
mTOR: mammalian target of rapamycin
ROS: reactive oxygen species
NAD^+^: nicotinamide adenine dinucleotide
PBMCs: peripheral blood mononuclear cells
PPARγ: peroxisome proliferator-activated receptor gamma
LPS: lipopolysaccharide
NRF: nuclear respiratory factor
NF-E2: nuclear factor (erythroid 2)-like
HO-1: heme oxygenase-1
SOD: superoxide dismutase
TFAM: mitochondrial transcription factor A
COX1: cytochrome c oxidase 1
COPD: chronic obstructive pulmonary disease
IGFBP3: Insulin-like growth factor binding protein 3
TNF: tumor necrosis factor
AECII: type II alveolar epithelial cells
PI3K: phosphatidylinositol 3 kinase
PTEN: phosphatase and tensin homolog deleted on chromosome 10
IKK: IκB kinase
MDM2: murine double minute 2
PIP3: phosphatidylinositol 3,4,5-trisphosphate
BALF: bronchoalveolar lavage fluid
Ang1: angiopoietin-1
MIP-2: macrophage inflammatory protein-2
STAT3: signal transducer and activator of transcription 3
EGR-1: early growth response protein 1
SP-1: specificity protein 1
ERK: extracellular signal-regulated kinase
MAPK: mitogen-activated protein kinase
JNK: c-Jun N-terminal kinase
MCP-1: monocyte chemoattractant protein-1
RAPTOR: regulatory associated protein of mTOR
ULK1: unc-51 like kinase 1
p70S6K: ribosomal S6 kinase
TSC: tuberous sclerosis protein
Rheb: ras homolog enriched in brain
LKB1: liver kinase B1
NAMPT: nicotinamide phosphoribosyltransferase.
